# Improved Bone Morphogenetic Protein-2 Retention in an Injectable Collagen Matrix Using Bifunctional Peptides

**DOI:** 10.1371/journal.pone.0070715

**Published:** 2013-08-08

**Authors:** Paul T. Hamilton, Michelle S. Jansen, Sathya Ganesan, R. Edward Benson, Robin Hyde-DeRuyscher, Wayne F. Beyer, Joseph C. Gile, Shrikumar A. Nair, Jonathan A. Hodges, Hanne Grøn

**Affiliations:** 1 Department of Microbiology, North Carolina State University, Raleigh, North Carolina, United States of America; 2 Affinergy, LLC, Research Triangle Park, North Carolina, United States of America; 3 Platform Technology and Science, GlaxoSmithKline, Research Triangle Park, North Carolina, United States of America; 4 Manufacturing Sciences, Biogen Idec, Research Triangle Park, North Carolina, United States of America; 5 QNS Group, LLC, Durham, North Carolina, United States of America; 6 Gile Surgical Support, Bangor, Maine, United States of America; 7 Haemophilia Biochemistry, Novo Nordisk, Måløv, Denmark; University of Milan-Bicocca, Italy

## Abstract

To promote healing of many orthopedic injuries, tissue engineering approaches are being developed that combine growth factors such as Bone Morphogenetic Proteins (BMP) with biomaterial carriers. Although these technologies have shown great promise, they still face limitations. We describe a generalized approach to create target-specific modular peptides that bind growth factors to implantable biomaterials. These bifunctional peptide coatings provide a novel way to modulate biology on the surface of an implant.

Using phage display techniques, we have identified peptides that bind with high affinity to BMP-2. The peptides that bind to BMP-2 fall into two different sequence clusters. The first cluster of peptide sequences contains the motif W-X-X-F-X-X-L (where X can be any amino acid) and the second cluster contains the motif F-P-L-K-G. We have synthesized bifunctional peptide linkers that contain BMP-2 and collagen-binding domains. Using a rat ectopic bone formation model, we have injected rhBMP-2 into a collagen matrix with or without a bifunctional BMP-2: collagen peptide (BC-1). The presence of BC-1 significantly increased osteogenic cellular activity, the area of bone formed, and bone maturity at the site of injection. Our results suggest that bifunctional peptides that can simultaneously bind to a growth factor and an implantable biomaterial can be used to control the delivery and release of growth factors at the site of implantation.

## Introduction

Approximately 7.9 million fractures occur each year in the United States alone, and approximately 10% of fractures exhibit delayed or impaired healing [1]. Bone morphogenetic proteins (BMPs) are osteogenic growth factors that have been shown to stimulate new bone formation and fracture healing [2], [3]. In clinical trials, recombinant human BMP-2 (rhBMP-2) has been shown to accelerate healing of open tibial fractures [4], and rhBMP-7 has been used to treat tibial nonunions [5]. These clinical applications, however, require open surgical procedures to insert the BMP–loaded carrier. In addition, supraphysiological amounts of BMPs are required to promote bone formation due to the growth factor's rapid diffusion away from its carrier [6], [7]. The use of high doses, however, raises concerns about bone formation away from the site and impact on nearby tissues and organs [8]; in accordance, rhBMP-2 use has been linked to a variety of serious adverse events [9].

Ideally, an injectable BMP-2 matrix carrier should have the following features: strong affinity for BMP to maintain biologically relevant concentrations over time to encourage osteoprogenitor cell migration, proliferation and differentiation; biocompatibility to minimize inflammation; sufficient porosity to allow cellular invasion and attachment; resorbability so that it will be replaced with new bone during healing; and appropriate viscosity for passage through a syringe without being washed away from the site of injection [10], [11], [12]. The carriers that have been explored for delivery of BMP include naturally derived polymers such as collagen, hyaluronic acid, chitosan, and fibrin; synthetic polymers such as polylactic acid (PLA), polyglycolic acid (PGA) and their copolymers (PLGA); ceramic materials including calcium phosphate cements; and various combinations of these materials [13]. For injectable BMP carriers, tested matrices include hyaluronan gels, gelatin (collagen) foams, composites of the gels and foams with tricalcium phosphate, and calcium phosphate cement [12]. Most of these injectable BMP carriers were unable to retain BMP at the site of injection; the carriers lost 50% or more of pre-loaded BMP after a few days *in vivo*. Calcium phosphate cement–based formulations were able to retain measurable amounts of BMP at the site of injection for 14 days [12]. These formulations, however, relied on entrapment of the BMP within the cement, raising concerns about a lack of release of BMP to the surrounding site of healing. We propose that the use of engineered peptides with high affinities for both BMP and the matrix material joined as a bifunctional peptide may provide more controlled release of BMP from the matrix and promote optimal healing *in vivo*.

Bifunctional peptides have been constructed by a number of groups as a way to modify materials and promote cell adhesion or growth factor binding. Gama and his colleagues [14], [15] have fused cell adhesion domains such as RGD and IKVAV to a cellulose-binding domain. The 25 kD fusion proteins promote fibroblast or mesenchymal stem cell adhesion to bacterial cellulose. Murphy and colleagues have also reported the successful use of modular peptides to deliver growth factors and mesenchymal stem cells to hydroxyapatite coatings [16], [17], [18].

In this study, we have used phage display technology to identify a series of peptides which bind to BMPs. The BMP–binding peptides can be organized into two sequence motifs. Consensus peptides from each motif were coupled to a collagen-binding peptide to form bifunctional peptides that can bind simultaneously to BMP-2 and collagen. The bifunctional peptide therefore can bind and retain BMP-2 onto a collagen matrix, slowing the release of BMP-2 from the matrix. We tested the ability of the bifunctional peptide to improve bone formation *in vivo* using a rat ectopic bone formation bioassay.

## Materials and Methods

### Ethics statement

All procedures with animals were performed under protocols approved by Affinergy's Institutional Animal Care and Use Committee in a facility with assurance from the Office of Laboratory Animal Welfare (A4544-01).

### Materials

Horseradish peroxidase (HRP)–conjugated anti-M13 monoclonal antibody was from GE Healthcare (Piscataway, NJ). Tween 20, 2,2′-Azino-bis(3-ethylbenzothiazoline-6-sulfonic acid) diammonium salt (ABTS), streptavidin (SA) from *Streptomyces avidinii*, bovine serum albumin (BSA), *para*-nitrophenylphosphate reagent (*p*-NPP) and all other chemicals were purchased from Sigma-Aldrich (St. Louis, MO). Recovered human plasma was purchased from the American Red Cross (Durham, NC). rhBMP-2 (355-BM/CF) and an anti-BMP antibody (MAB3552) were purchased from R&D Systems (Minneapolis, MN). *In vivo* studies were performed using rhBMP-2 (INFUSE) purchased from Medtronic (Ref. 7510600). N-α-Fmoc-amino acids (with orthogonal side chain protecting groups) were purchased from Novabiochem (Merck KGaA, Darmstadt, Germany). Alkaline phosphatase–labeled goat anti-mouse secondary antibody was purchased from Promega (Madison, WI).

### Phage Display

rhBMP-2 was biotinylated using a Sulfo-NHS-Biotin reagent (Pierce EZ-link biotinylation kit) following the manufacturer's protocol. The biotinylated rhBMP-2 was immobilized onto a streptavidin-coated 96-well microtiter plate (Immulon IV) and the plates blocked with 0.5% BSA in phosphate buffered saline, 0.05% Tween-20 (PBST). Phage display was performed as previously described [19], [20]. Ten different phage display libraries were screened for peptides that bind to rhBMP-2. Each library was designed around a specific amino acid motif or amino acid bias. After 3 rounds of phage display selections, the pools of enriched phage were plated on a lawn of *E. coli* DH5αF' cells. Individual phage were picked and propagated on *E. coli* overnight. The cells were removed by centrifugation, and 10 μl of the phage-containing supernatant was added to the wells containing BMP-2 or to a control well containing buffer. After incubation and washing, phage were detected in an ELISA–type assay using an HRP-conjugated, anti-M13 monoclonal antibody (1∶1000).

### Generating Focused Library for BMP-2 binding peptides

To generate the Motif 1–focused and Motif 2–focused libraries, oligonucleotides were synthesized to encode peptides that have a restricted set of possible amino acids in selected positions in the peptide (**Table 1**). A short complementary primer was annealed to the 3′ end of the library oligonucleotide and extended with T7 polymerase. After second strand synthesis, the DNA was digested with Xba I and Xho I and ligated into mAEK phage display vector. The ligation reaction was used to transform electro-competent *E. coli* DH12S. Transformed cells were grown overnight in 2×YT medium. The phage-containing culture supernatant was collected and the phage concentrated by PEG precipitation. Precipitated phage were resuspended in phosphate buffered saline containing 20% glycerol, aliquoted and stored at −20°C.

**Table 1:** Focused library design for motif 1 and motif 2 BMP-binding peptides*.

Motif 1 Library	X_5_-[W/L/C/Y/F/S]-X_2_-[W/L/C/Y/F/S]-X-[A/G/N/S/T]-[L/F/I/M/V]-X_5_
Motif 2 Library	X_3_-[L/F/I/M/V]-X-[W/L/C/Y/F/S]-[P/S/T/A]-[ L/F/I/M/V]-[I/M/T/N/K/S/R]-X_8_

* X can be any of the 20 natural amino acids. Amino acids in brackets [ ] are the restricted set of amino acids allowed in that position in the encoded peptide library.

### DNA sequence analysis

DNA from isolated positive phage clones was amplified using the TempliPhi DNA amplification kit and DNA analysis was performed by Sequetech (Mountain View, CA).

### Peptide Synthesis

Peptides were synthesized by solid-phase peptide synthesis techniques on a Rainin Symphony Peptide Synthesizer using standard Fmoc chemistry (HBTU/HOBT activation, 20% piperidine in DMF for Fmoc removal). After all residues were coupled, simultaneous cleavage and side chain deprotection was achieved by treatment with a trifluoroacetic acid (TFA) cocktail. Crude peptide was precipitated with cold diethyl ether and purified by high-performance liquid chromatography on a Shimadzu HPLC using a Vydac C18 reversed-phase silica column (10 μm, 120 Å, 250 mm ×22 mm) using a linear gradient of water/acetonitrile containing 0.1% TFA. Homogeneity of the synthetic peptides was evaluated by analytical RP-HPLC (Vydac C18 silica column, 10 μm, 120 Å, 250 mm ×4.6 mm) and the identity of the peptides was determined by MALDI-TOF-MS. All peptides were synthesized with a biotin coupled to the epsilon-amino group of the C-terminal lysine.

### Bifunctional Peptide Design

A consensus BMP-binding peptide was generated for each BMP-binding motif. Peptides that bind to collagen were isolated by phage display on demineralized bone matrix [21]. Bifunctional peptides (Collagen: BMP) were generated by joining a collagen-binding peptide sequence to each of the BMP-binding sequences through a short, flexible amino acid linker (GSSG): [peptide BC-1] – SWWGFWNGSAAPVWSR-GSSG-AGAWEAFSSLSGSRV-GSSGK(Biotin) and [peptide BC-2] – SWWGFWNGSAAPVWSR-GSSG-AGALGFPLKGEVVEGWA-GSSGK(Biotin). The GSSG linker is intended to join the two binding domains and has no inherent properties.

### BMP-2 peptide binding assay

To measure the ability of a BMP-binding peptide to capture its target growth factor out of buffer or a complex biological solution such as plasma, biotinylated peptides were immobilized onto a streptavidin-coated 96 well plate. Varying low nanomolar concentrations of BMP-2 were “spiked” into human plasma or Tris-buffered saline (0.5 M NaCl), 0.05% Tween-20 (TBST) and added to the peptide-containing plates. After 1 hr incubation at room temperature (RT), plates were washed and incubated with an anti-BMP-2 antibody followed by a goat anti-mouse secondary antibody conjugated to alkaline phosphatase. Adding *p*-NPP produced a colored reactant, which was quantified using a SpectroMax (Molecular Devices) plate reader at 405 nm.

### Preparation of a 4% fibrillar collagen gel

Collagen stock solution (6.4 mg/ml, Inamed cat#5413, lot1387646) was neutralized overnight at room temperature using 200 mM sodium phosphate, pH 9.4. The fibrillar collagen was pelleted by centrifugation at 17,200×g for 20 minutes at 10°C. The collagen weight percent was determined using a bicinchoninic acid protein assay (BCA assay, Pierce cat#23255).

### Preparation of 1.5% injectable collagen gel for BMP-2 delivery *in vivo*

The stock fibrillar collagen (4%) was diluted in PBS containing rhBMP-2 with or without the collagen-BMP bifunctional peptide, to generate a final collagen wt% of 1.5%. The amount of BMP-2 and peptide were optimized using pilot experiments *in vivo* such that 200 µl of the injected collagen gel contained 2 µg of BMP-2 and a 50-fold molar excess of the peptide.

### Injectable collagen gel binding assay

The injectable collagen gel (0.1 mL) was aliquoted into a polypropylene plate. The plate was blocked with 150 µl of 1% BSA in TBS for 30 min. After spinning for 2 min, the supernatant was removed without disrupting the collagen gel. The bifunctional peptide (30 μM) was mixed with rhBMP-2 (0.0017 to100 nM) in Binding Buffer (125 mM glutamic acid, 10% sucrose, 12.5% glycine, 10% polysorbate 80, 5 M NaCl) for 30 min with gentle agitation. The peptide: rhBMP-2 complex (50 μL) was then added to the collagen gel and incubated at room temperature for 1 h with gentle agitation. The collagen gel was washed three times with TBST and rhBMP-2 was detected with an anti-BMP antibody (R&D Systems MAB3552). The wells were then washed, and an alkaline phosphatase labeled goat anti-mouse secondary antibody was added. After incubation and washing, binding was measured using the chromogenic reagent *p*-NPP, and absorbance was read at 405 nm.

### *In vivo* ectopic bone formation study

Male Sprague Dawley rats (150–175 g) were obtained from Taconic farms, Charles River Laboratories (Raleigh, NC) and acclimated prior to surgery. The surgery was performed under general anesthesia by weight-adapted intraperitoneal injection of Xylazine 2% (Medistar; 12 mg/kg body weight) and Ketamine hydrochloride (Ketaset; 100 mg/mL; 80 mg/kg body weight). The thorax and abdomen were shaved and scrubbed with Betadine and alcohol. Using aseptic technique, two 5 mm incisions were made at the midline so that two subcutaneous pouches were prepared by blunt dissection. Collagen gel (200 µl) containing 2 µg rhBMP-2 with or without collagen-BMP bifunctional peptide (BC-1) was injected into the left or the right side of the subcutaneous region. Each rat received two injections and a total of 20 animals were used in the study. The BMP-2 dose (2 μg) used in this experiment had been determined from preliminary experiments (data not shown). In those experiments, rats were implanted with collagen gel containing either 1 or 5 μg of BMP-2. Collagen gel without peptide resulted in bone formation at the 5 μg dose but not at the 1 μg dose. Collagen gel with peptide resulted in bone formation at both doses (1 and 5 μg), and a dose of 2 μg BMP-2 was selected for the experiment.

The sites containing the injected material were explanted at two weeks and fixed in 10% neutral buffered formalin. The samples were placed in Formical 2000 decalcifier (American Mastertech) for 24 hours followed by Cal-arrest neutralizing solution for one hour. The tissue samples were processed in a Thermo-Electron, Shandon Excelsior Automated Tissue Processor for 14 hours. The process includes additional fixation in 10% buffered neutral formalin, dehydration through an ethanol gradient, and clearing with xylene at 40°C. Tissue was then embedded on a Leica EF 1140 H tissue embedding center in paraffin blocks for sectioning. Paraffin blocks were sectioned at 5 microns on a Reichert-Jung RM2065 microtome using Accu-Edge High Profile disposable stainless steel microtome blades. Three serial sections at intervals of 30 microns were obtained and placed on Super-frost Plus slides for hematoxylin and eosin staining (H&E). Staining was performed using a Sakura DRS-601 automatic slide stainer with a regressive Harris hematoxylin and Eosin Y Alcoholic for histological and morphological examination.

Histological sections were scored based on three criteria: Bone Cross-Sectional Area, Bone Maturity and Cellular Activity. Each segment was scored based on a 0–4 point scale by two observers (blinded to the study) who evaluated the entire implant under 10× magnification. The scoring system is summarized in **Table 2**.

**Table 2:** Scoring system for histological analysis of bone growth.

GRADE	BONE CROSS-SECTIONAL AREA*
0	No Evidence of Bone Formation
1.1	1–10% of Implant Shows Evidence of Bone Formation
1.2	11–25% of Implant Shows Evidence of Bone Formation
2.1	26–35% of Implant Shows Evidence of Bone Formation
2.2	36–50% of Implant Shows Evidence of Bone Formation
3	51–75% of Implant Shows Evidence of Bone Formation
4	76–100% of Implant Shows Evidence of Bone Formation

GRADE	BONE MATURITY
0	No Bone
1	Immature/Unorganized
2	Immature
3	Mature
4	Mature/Well Organized

GRADE	CELLULAR ACTIVITY
0	None
1	Rare
2	Few
3	Moderate
4	Dense

* Implant evaluated at 10× Magnification.

## Results

### Isolation of BMP-binding peptides using phage display peptide libraries

**Figure 1:**
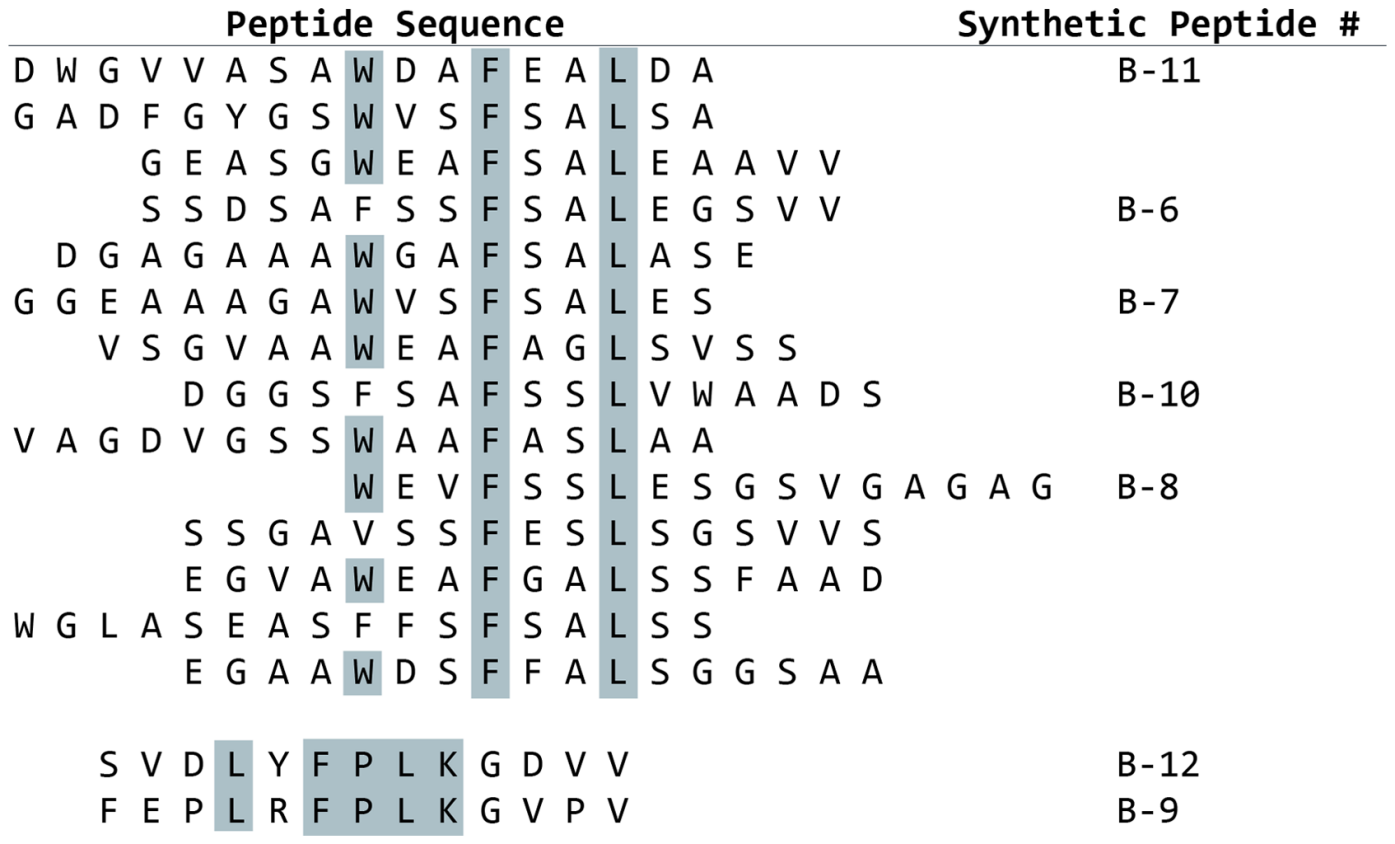
BMP-2 Binding Peptides. Biotinylated BMP-2 was immobilized on streptavidin-coated plates and subjected to multiple rounds of phage display selections using 10 different phage display peptide libraries. Individual BMP-binding phage were isolated and the sequence of the BMP-binding peptide deduced from the phage DNA sequence. Alignment of the peptides revealed two general sequence motifs among the peptides: motif 1: W-X-X-F-X-X-L and motif 2: L-X-F-P-L-K. These motifs were used to generate 2^nd^ generation focused libraries. In addition, representative synthetic peptides were made and tested for binding to BMP-2.

Phage display was performed against immobilized rhBMP-2 with 10 different peptide libraries, representing over 10 billion peptide sequences. Biopanning with these libraries revealed a set of 16 peptides that bound to BMP-2 (**Figure 1**). These first-generation BMP–binding peptides fall into two different sequence clusters. The first cluster of peptide sequences contains the motif W-X-X-F-X-X-L (single letter amino acid code, where X can be any amino acid), designated motif 1, and the second cluster contains the motif F-P-L-K-G, designated motif 2. A series of truncations in which conserved amino acids were deleted in the BMP-binding peptides results in a loss of BMP-2 binding activity (data not shown). The consensus sequence among the peptides indicates that all the peptides within a sequence cluster are binding to the same site on BMP-2, and the common amino acids that make up the motif are responsible for the specific interactions with BMP-2.

**Figure 2:**
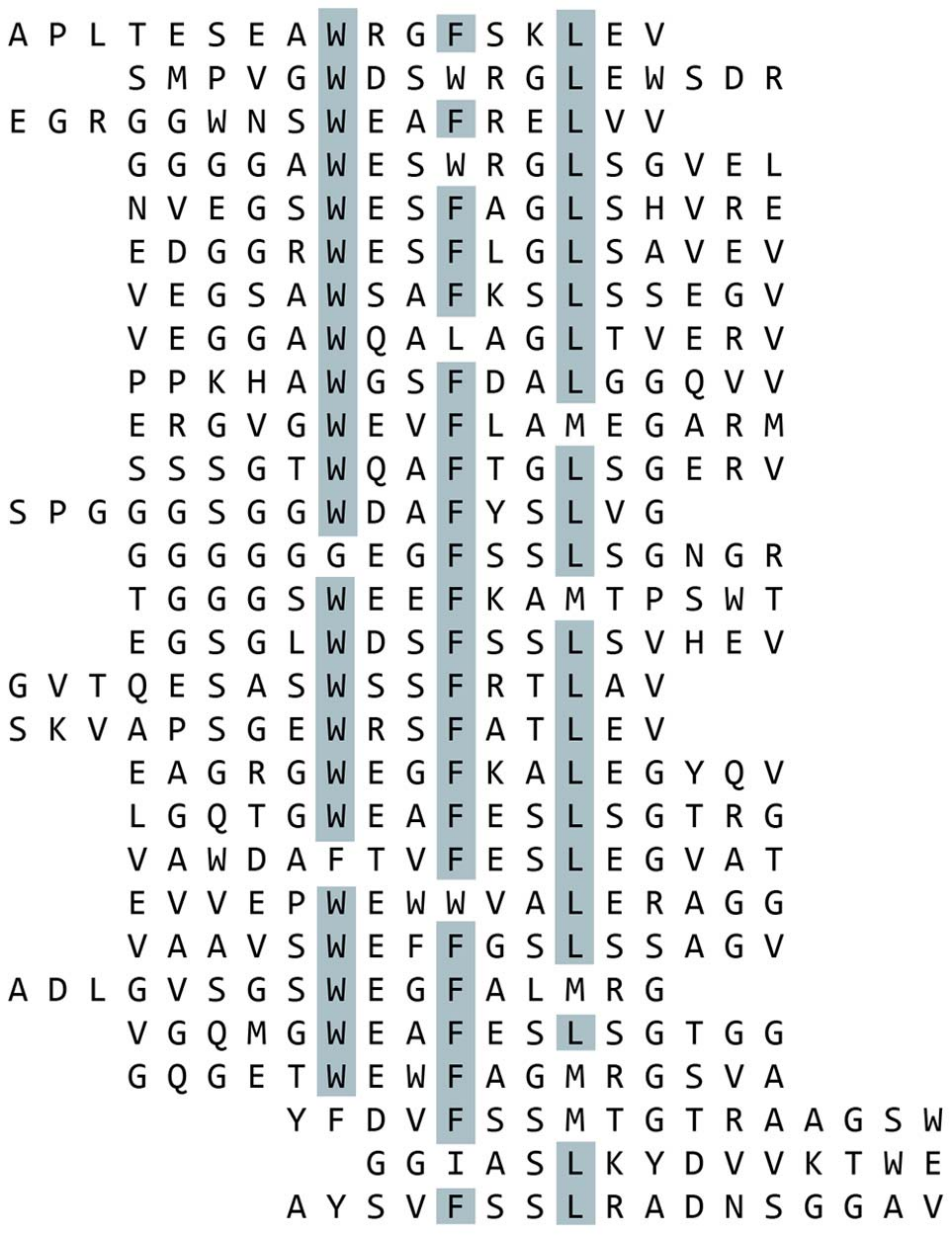
BMP-2 Binding Peptides Containing Motif 1 Isolated from Selections Using the Focused Libraries. Biotinylated BMP-2 was immobilized on streptavidin-coated plates and subjected to multiple rounds of phage display selections using the focused libraries. Peptides which contain motif 1 were aligned. Amino acids which are present in over half of the aligned sequences at a given position are highlighted.

**Figure 3:**
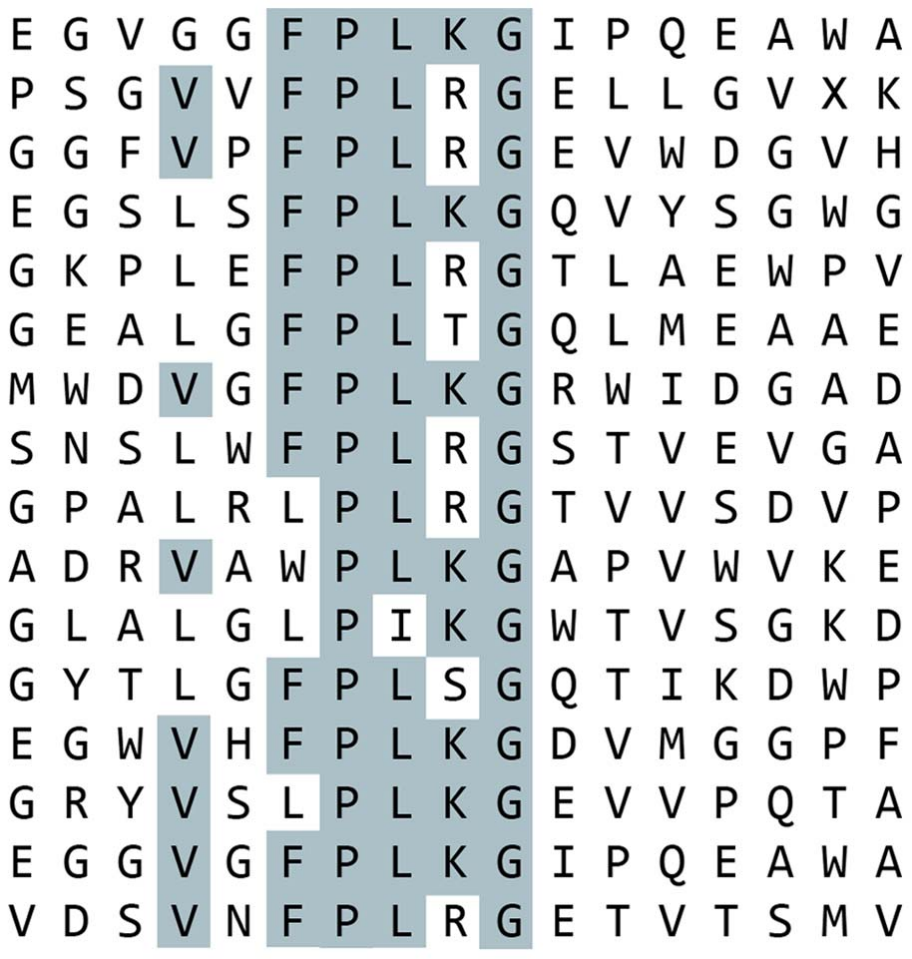
BMP-2 Binding Peptides Containing Motif 2 Isolated from Selections Using the Focused Libraries. Biotinylated BMP-2 was immobilized on streptavidin-coated plates and subjected to multiple rounds of phage display selections using the focused libraries. Peptides which contain motif 2 were aligned. Amino acids which are present in over half of the aligned sequences at a given position are highlighted.

Using these sequence motifs, we designed a focused phage display library around each motif (**Table 1**). Each of the focused libraries was screened for additional peptide sequences that would bind to BMP-2 using standard phage display techniques [22]. Combined, 59 peptide sequences were found positive for BMP-2 binding. Of these 59, 41 sequences were found around motif #1 (**Figure 2**) and 18 represented motif #2 (**Figure 3**). After aligning the peptides, the frequency of amino acid identity was scored at each position (**Tables 3** **&** **4**). Positions that have only one, two or three amino acid identities represented at a given position are likely to promote BMP–binding, therefore defining a new feature for the binding motif. From the alignment, a consensus sequence was established for the binding domain revealed by each focused library. Synthetic peptides were synthesized based on each consensus sequence: B-17 is the consensus for motif #1 and B-18 is the consensus for motif #2 (**Table 5**).

**Table 3:** Summary of BMP-2 binding peptides containing motif 1*.

G3	G4	G7	G11	G13	G15	A15	W33	E18	A14	F37	S13	A14	L36	S16	G16	S6	R6	V12
A2	L3	V6	E7	S8	A5	S10	F5	D6	S14	W3	A7	S14	M5	E11	S7	A5	G5	A4
D2	V2	E5	V5	A5	S5	G8	G1	S6	V5	L1	E5	G8		A3	V7	E4	V5	G3
P2	D1	A4	A4	E2	V4	T2	V1	G2	G4		R4	T2		R3	A6	V4	A3	R2
K1	E1	S4	S4	Q2	E3	E1	Y1	Q2	W2		K3	E1		T3	W2	T3	E3	T2
V1	R1	T2	D2	V2	D1	L1		R2	E1		G2	K1		V3	H1	G2	S3	D1
W1	T1	D1	P2	W2	H1	P1		V2	F1		L2	L1		D1	P1	D1	D1	E1
		F1	M1	D1	M1	R1		A1			D1			G1	R1	F1	M1	L1
		L1	Q1	K1	N1	Y1		F1			F1					N1	Q1	M1
		N1	R1	P1	R1			T1			T1					P1	W1	S1
		P1		Y1	T1						V1					Y1		
		Q1									Y1							

* Based on 41 sequences. Format is single-letter amino acid code and frequency of occurrence at that position in the peptide.

**Table 4:** Summary of BMP-2 binding peptides containing motif 2*.

G7	G5	A3	L9	G6	F14	P18	L17	K10	G18	E4	V6	V8	E5	G5	W4	A4
E4	D2	S3	V8	R2	L3		I1	R6		Q3	P4	I2	S3	A3	A2	D2
S2	E2	G2	G1	S2	W1			S1		D2	T4	M2	D2	V3	K2	E2
A1	K1	D2		A1				T1		I2	L3	Q2	G2	D2	P2	P2
F1	L1	P2		E1						T2	W1	A1	K1	Q1	V2	V2
M1	N1	F1		H1						A1		L1	P1	T1	G1	H1
P1	P1	R1		N1						R1		W1	T1	W1	M1	K1
V1	R1	T1		P1						S1		Y1	W1		T1	F1
	S1	V1		V1						V1						G1
	V1	W1		W1						W1						
	W1	Y1		Y1												
	Y1															

* Based on 18 sequences. Format is single-letter amino acid code and frequency of occurrence at that position in the peptide.

**Table 5:** Sequence of consensus BMP-binding peptides.

Synthetic Peptide #	Peptide Sequence
B-17 (motif 1)	GGGAWEAFSSLSGSRVGSSGK-(Biotin)
B-18 (motif 2)	GGALGFPLKGEVVEGWAGSSGK-(Biotin)

### Relative affinity of BMP-2 binding peptides for BMP-2

**Figure 4:**
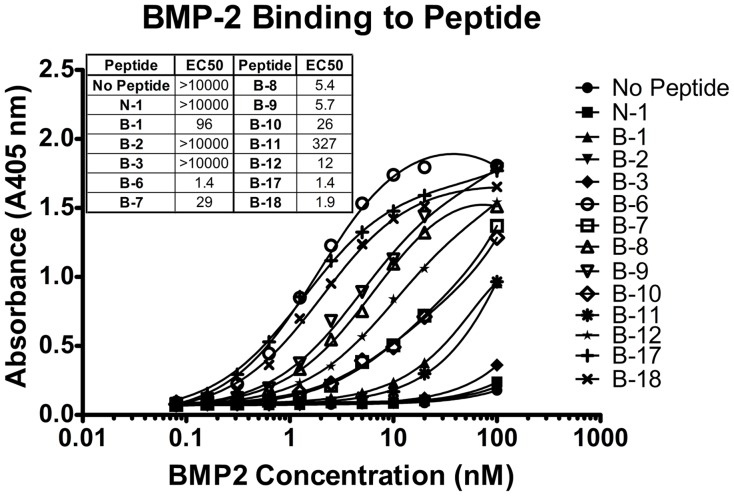
BMP-2 Binding to Peptide. Biotinylated peptides were immobilized on streptavidin-coated plates and incubated with a range of rhBMP-2 concentrations in TBST for 1 h. BMP-2 binding was analyzed using antibody and *p*-NPP detection. Peptides B-6, B-17, and B-18 had the highest binding affinities for rhBMP-2. Peptide N-1 is a negative control that binds hexokinase. Data are presented as the absorbance read at 405 nm.

To compare binding affinities between first and second generation BMP–binding peptides, we tested a range of BMP-2 concentrations (0.001 to 10 pmoles/well) for binding against immobilized peptide on streptavidin coated plates (**Figure 4**). The consensus peptides B-17 and B-18 bound BMP-2 with high apparent affinity with EC50 values of 1.4 and 1.9 nM, respectively. Peptide B-6 also had a low EC50 value of 1.4 nM but was not used in subsequent experiments.

### Cross-reactivity of BMP-2–binding peptides with other growth factors

**Figure 5:**
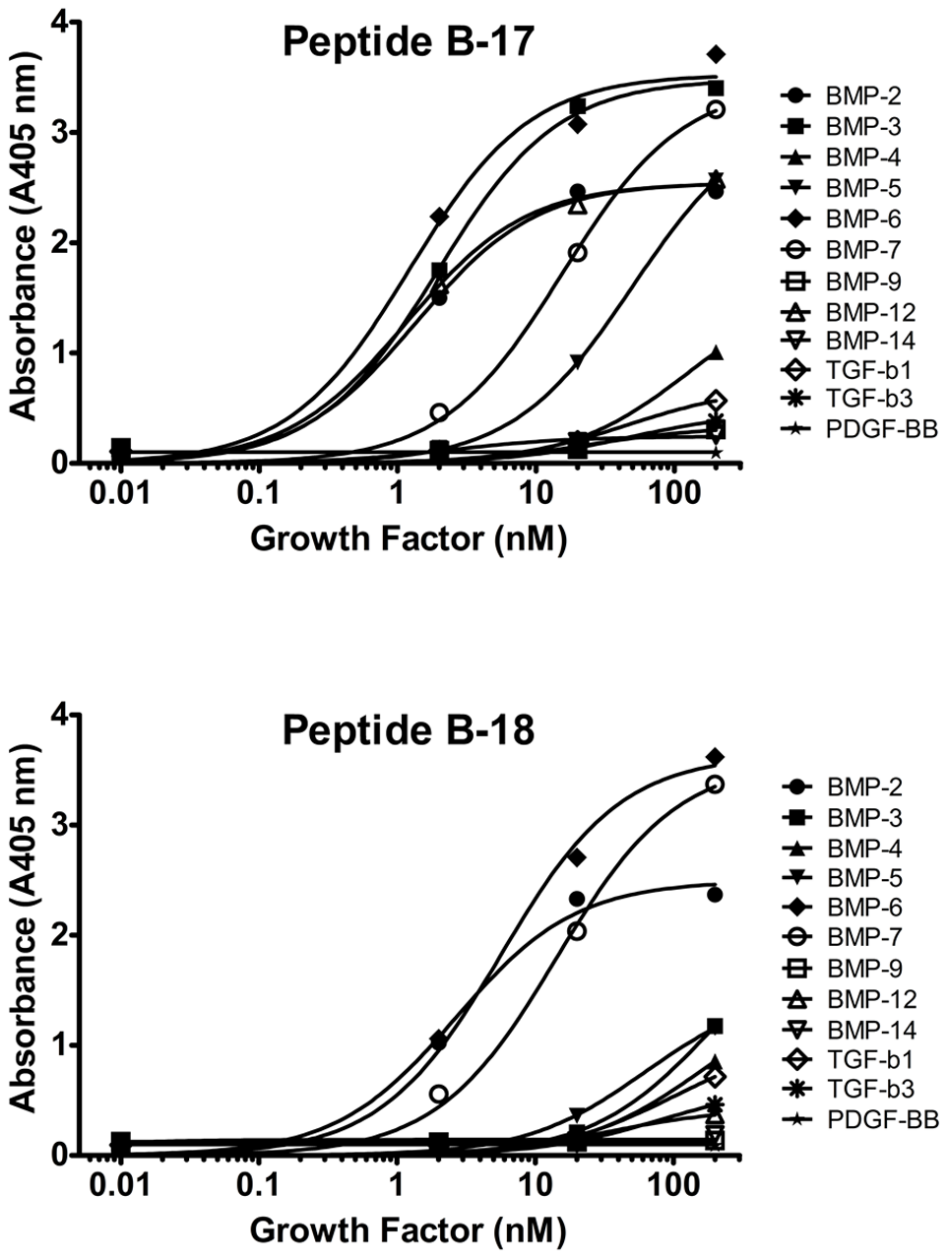
Peptide cross-reactivity to other growth factors. Peptides B-17 (A) and B-18 (B) were immobilized on a streptavidin-coated plate, and a range of concentrations of growth factors in the TGF-β superfamily were titrated onto the plate in TBST for 1 h. Growth factor binding was analyzed using antibody and *p*-NPP detection. Both peptide B-17 and B-18 bound to rhBMP-2, rhBMP-6, and rhBMP-7. Peptide B-17 also cross-reacted with rhBMP-3, rhBMP-5, and rhBMP-12. The peptides had lower affinities for all other growth factors tested. Data are presented as the absorbance read at 405 nm.

BMPs are members of the TGF-β superfamily and so we examined our peptides' abilities to bind other family member proteins. The two BMP–binding peptides that contain the consensus motif #1 (B-17) and consensus motif #2 (B-18) were tested for binding to BMP-2, -3, -4, -5, -6, -7, -9, -12, -14, TGF-β1, TGF-β3, and PDGF-BB. Both peptides bound to BMP-2, BMP-6 and BMP-7 but showed no binding to TGF-β1, TGF-β3, or PDGF-BB **(** **Figure 5**; EC50, Peptide B-17: 1.4 nM, BMP-2; 1.3 nM, BMP-6; 17.0 nM, BMP-7. EC50, Peptide B-18: 2.8 nM, BMP-2; 6.5 nM, BMP-6; 16.2 nM, BMP-7**)**. The lack of specific binding for TGF-β1, TGF-β3 or PDGF-BB, suggests that the peptides intended for BMP-2 binding harbor a specific interaction with a sequence or structural motif found in several BMPs but not in other growth factors. The two peptides, B-17 and B-18, however, do not show identical specificity among the BMP proteins. B-17 also bound to BMP-3, BMP-5 and BMP-12, whereas B-18 did not. Competition experiments with B-17 and B-18 demonstrated that the two peptides compete for binding to BMP-2 (data not shown). This finding indicates that the peptides bind at or near the same site on BMP-2. The cross-reactivity results indicate that B-17 binds to features on BMPs that are found in BMP-2, -3, -5, -6, -7, and -12 whereas B-18 binds to features found in only BMP-2, -6, and -7.

### Capture of BMP-2 from a complex biological fluid

**Figure 6:**
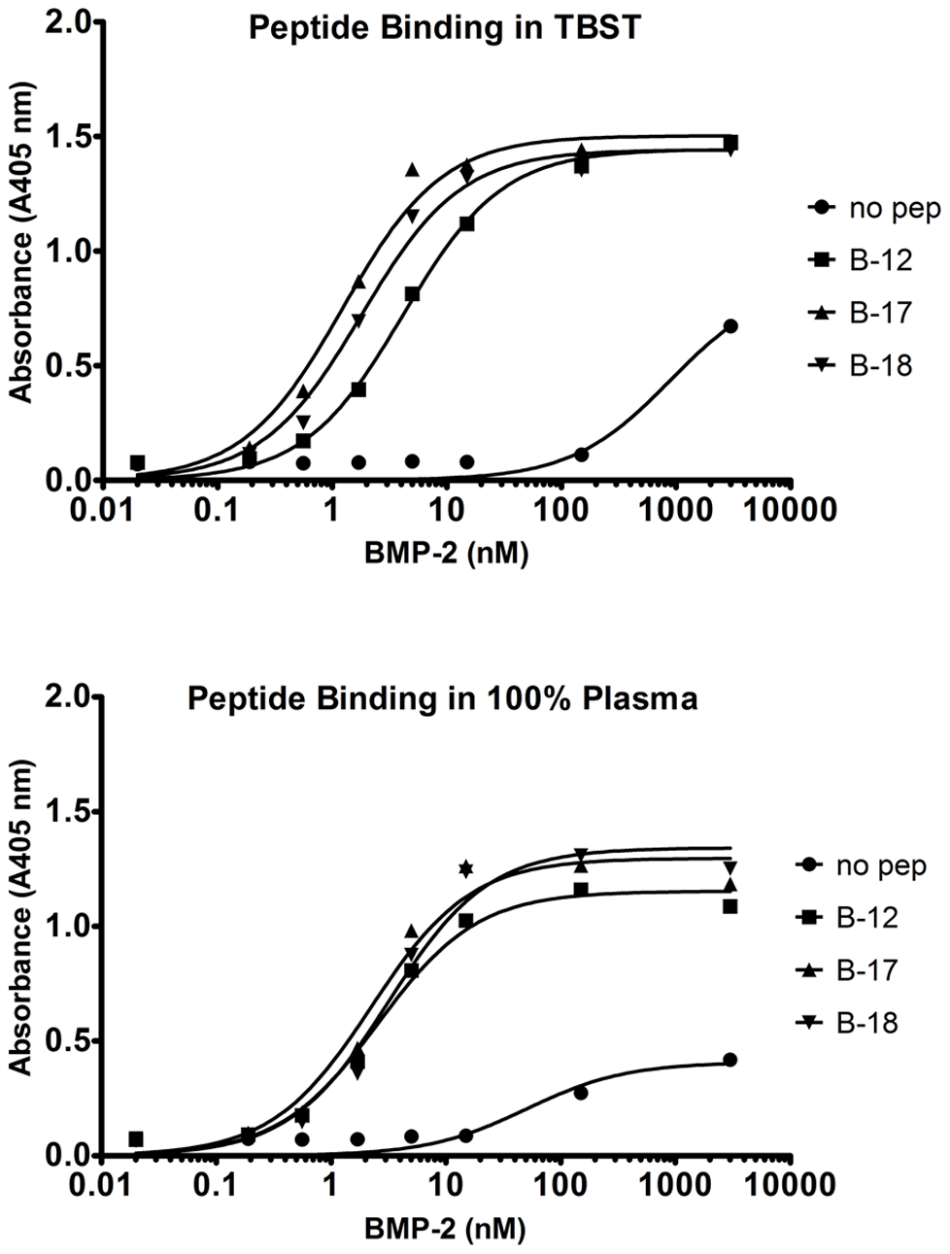
Peptide-mediated capture of BMP-2 from spiked TBST or plasma. The biotinylated peptides were coated on streptavidin plates and incubated in TBST (**A**) or plasma (**B**) with nanomolar concentrations of rhBMP-2 for 1 h. BMP-2 binding was analyzed using antibody and *p*-NPP detection. The peptides captured more rhBMP-2 from solution than the no peptide control. Data are presented as the absorbance read at 405 nm.

To model endogenous capture of a specific protein *in vitro*, picomole amounts of rhBMP-2 (0.001 to 15 pmoles/well) were mixed with TBST or human plasma for 1 h. After washing the plates in TBST, an alkaline phosphatase–conjugated antibody against BMP-2 was applied, and bound BMP-2 was measured with a colorimetric assay on a plate reader. Incubating BMP-2–binding peptides in human plasma had no effect on the peptide's ability to bind and capture BMP-2 (**Figure 6**). Binding reactions in TBST controls exhibited virtually identical affinity for the target growth factor relative to the biological fluids. These data show that the peptides are not “fouled” by other proteins in biological fluids, suggesting that bifunctional peptide–mediated capture and concentration of endogenously produced growth factors could also be a viable therapeutic strategy.

### Bifunctional Peptides

**Figure 7:**
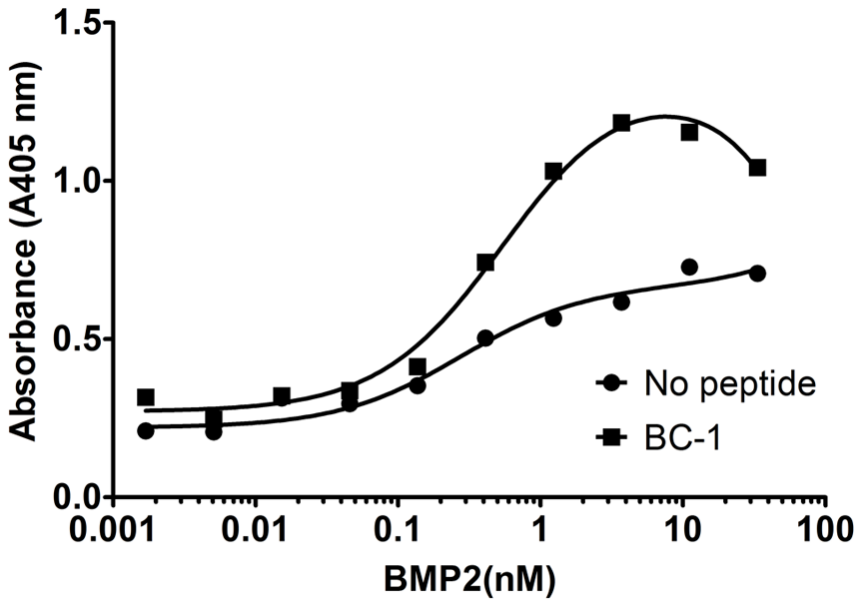
Bifunctional peptide–mediated binding of BMP-2 to injectable collagen. A bifunctional peptide (BC-1) was synthesized containing Peptide B-17 and a collagen-binding peptide with a short amino acid linker. rhBMP-2 was mixed with or without the bifunctional peptide and added to a collagen gel. The bifunctional peptide enhanced the retention of rhBMP-2 to the collagen gel (no peptide, EC50 = 5.5 nM; BC-1, EC50 = 0.41 nM). Data are presented as the absorbance read at 405 nm.

Bifunctional peptides consisting of the BMP–binding sequences and the collagen-binding sequences were synthesized and tested for the ability to bind BMP-2 to a collagen gel. Both BC-1 and BC-2 bound BMP-2, but to minimize the size and cost of the animal study, only BC-1 was used in the *in vivo* model. BC-1 was chosen because the BMP-binding sequence in BC-1, the B-17 sequence, bound a wider range of BMPs than the B-18 sequence which might be useful in future experiments. BMP-2 was incubated with the bifunctional peptide BC-1 and then added to plates containing the injectable collagen gel. BMP-2 is known to bind collagen weakly [23], but the bifunctional peptide increased binding of BMP-2 to the collagen gel more than 10-fold (no peptide: EC50 = 5.5 nM; bifunctional peptide: EC50 = 0.41 nM; **Figure 7**).

### *In vivo* model – Ectopic Bone Formation

**Figure 8:**
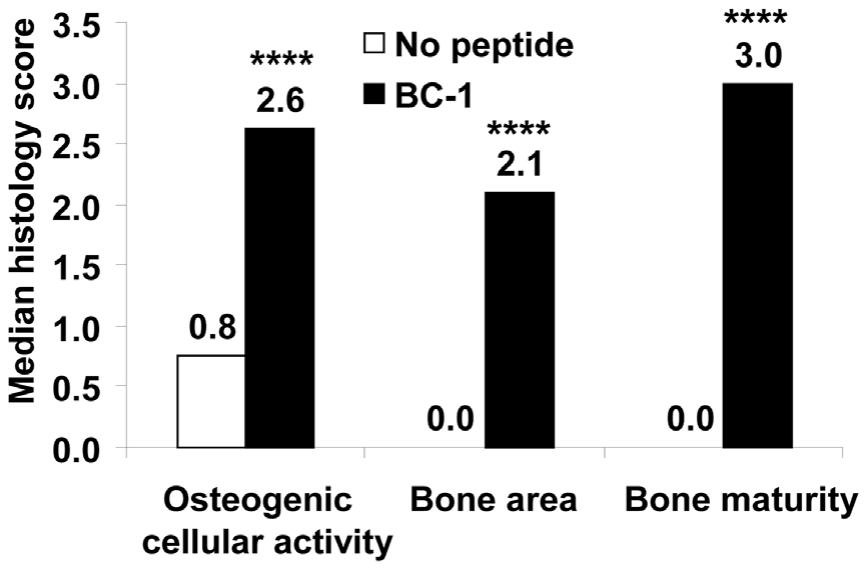
Ectopic bone formation with rhBMP-2 delivered in a collagen gel with or without the bifunctional peptide. rhBMP-2 (2 μg) was delivered in a rat ectopic bone model either alone (no peptide) or in combination with a 50-fold molar excess of the bifunctional peptide (Collagen-BMP peptide). H&E stained slides were scored for osteogenic cellular activity, bone area and bone maturity by two observers and the median score for each group is shown in the figure. ****, p<.0001 vs no peptide.

**Figure 9:**
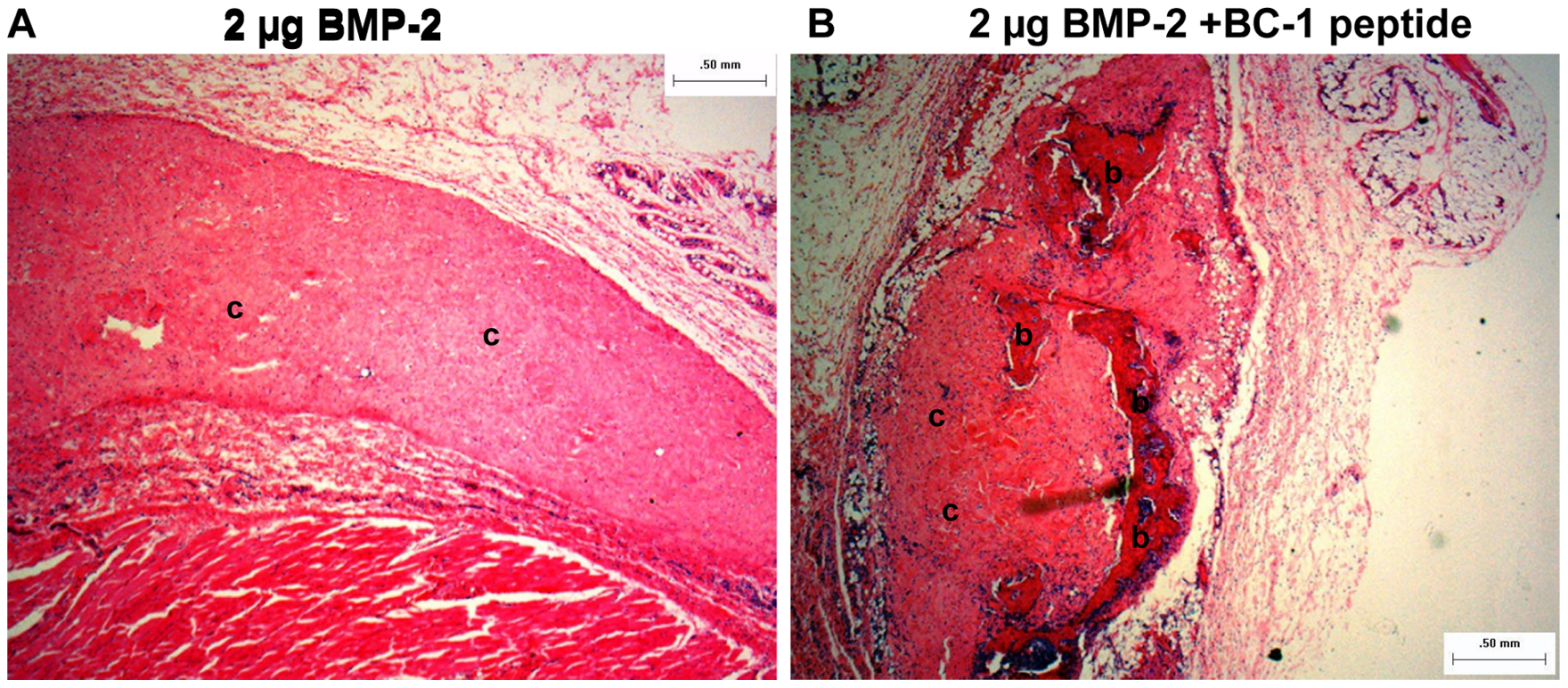
Representative histology image (hematoxylin and eosin stain) from the rat ectopic model obtained at a 2X magnification. **A**: 2 µg BMP-2 in 1.5% collagen gel and **B**: 2 µg BMP-2 with 50-fold molar excess of collagen-BMP-2 bifunctional peptide in 1.5% collagen gel. **b** – Represent regions of bone; **c** – represent regions of collagen; cells are stained blue. The image shows only cellular activity in sample A, whereas sample B shows bone formation and increased cellular activity.

Ectopic bone formation at two weeks was evaluated by histological scoring of the H&E stained slides by two observers. The slides were scored for infiltration of osteogenic cells (fibroblasts, osteoblasts, osteogenic progenitors and cells of the cartilage), each of which were scored on a scale of 1–4: 1-rare, 2-few, 3-moderate and 4- dense. Osteogenic cellular activity was obtained by an average of the scores for each cell type for every animal. Comparison between groups was performed using medians. As shown in **Figure 8**, the median cellular activity of the peptide group was significantly greater than the no peptide (BMP-2 alone) group (Mann-Whitney U two tailed test, p<0.0001). Bone area was scored as percent of the implant showing new bone formation on a scale of 0–4. The bone area was significantly greater in the peptide group compared to the no peptide (BMP-2 alone) group (Mann-Whitney U two tailed test, p<0.0001). In the peptide group, approximately 25% of the implant was covered with new bone, whereas no new bone was detected in the no peptide group. The maturity of the newly formed bone was scored on a scale of 0–4, with 0-no bone, 1-immature/unorganized, 2-immature, 3-mature and 4- mature/well-organized. In the absence of peptide very little and immature/unorganized bone was formed. Bone was formed with rhBMP-2 in the presence of the peptide and it was characterized as mature. Representative images obtained at a 2× magnification are shown in **Figure 9** and the histology scores (average of two observers blinded to the study) of all samples are presented in **Table 6**.

**Table 6:** Histology scores (average from two observers blinded to the study) of all samples with rhBMP-2 delivered in a collagen gel with or without the bifunctional peptide.

Osteogenic Cellular activity		Bone area		Bone maturity	
2 µg rhBMP-2	2 µg rhBMP-2+ bifunctional peptide	2 µg rhBMP-2	2 µg rhBMP-2+ bifunctional peptide	2 µg rhBMP-2	2 µg rhBMP-2+ bifunctional peptide
0.8	2.8	0.0	2.1	0.0	3.0
0.8	3.0	0.0	2.2	0.0	3.0
0.8	3.0	0.0	2.1	0.0	3.0
0.8	3.0	0.0	2.2	0.0	3.0
0.8	2.0	0.0	1.1	0.0	2.0
0.5	3.8	0.0	3.0	0.0	3.0
0.5	2.6	0.0	2.5	0.0	3.0
1.8	2.5	0.0	2.1	0.0	2.0
1.8	3.1	1.1	2.5	1.0	3.0
0.5	1.8	0.0	0.0	0.0	0.0
1.5	2.4	0.0	1.2	0.0	2.0
0.8	2.5	0.0	1.2	0.0	2.0
0.8	2.5	0.0	1.2	0.0	3.5
0.8	3.3	0.0	2.2	0.0	3.0
0.8	*nd	0.0	*nd	0.0	*nd
1.5	2.4	0.0	1.1	0.0	2.0
0.8	2.6	0.0	1.2	0.0	2.0
1.3	1.9	0.0	0.0	0.0	0.0
0.8	3.5	0.0	3.5	0.0	3.5
1.3	1.5	0.0	0.0	0.0	0.0

* nd –not determined.

## Discussion

Using phage display technology and biopanning on rhBMP-2, we have identified a set of peptides that bind to several forms of BMP. The peptides can be organized into two groups based on common elements in their peptide sequences. The first group shares the sequence motif W-X-X-F-X-X-L (motif 1) and the second group shares the sequence motif F-P-L-K-G (motif 2). The forty sequences that comprise motif 1 all bind to BMP at a common site and the conserved amino acids in motif 1 are largely responsible for the binding of those peptides to BMP. Similarly, the eighteen peptides that group into motif 2 bind at a common site on BMP and the binding is determined by the conserved amino acids in motif 2. Aligning all the peptides in motif 1 or motif 2 and using the most frequently identified amino acid in each position of the peptide allowed a consensus binding sequence to be generated for each motif. The consensus peptides B-17 and B-18 showed strong binding to BMP-2 with EC50 values around 1–2 nM. Although neither of these sequences was isolated in the phage display selection on BMP-2, the aggregation of the peptide sequences from the selections led to the design of peptides B-17 and B-18, which have high affinity for the target protein.

To use the BMP-binding peptides in a drug delivery system, we linked the consensus BMP-binding peptide (B-17) to a previously identified collagen-binding peptide through a short flexible four amino acid linker. The bifunctional peptide maintained the activity of each peptide domain: collagen binding and BMP-2 binding. *In vitro*, the bifunctional peptide BC-1 was able to increase binding of BMP-2 to a collagen gel by 10-fold. *In vivo*, ectopic bone formation was analyzed in rats using an injectable 1.5% collagen gel containing 2 μg of rhBMP-2 with or without BC-1. The concentration of injected collagen gel (1.5% w/w), BMP-2 dose (2 µg), peptide to BMP-2 molar ratio (50∶1) and study duration (2 weeks) were optimized from preliminary experiments (data not shown). The presence of BC-1 significantly increased osteogenic cellular activity, the area of bone formed, and bone maturity.

To increase osteogenic activity of cells, BMP-2 has to interact with the BMP receptors on the cells. From our experiments, it is not possible to distinguish whether BMP-2 can bind the receptor while still bound to the peptide or if the BMP-2 is released from the peptide and then interacts with the BMP receptor. In cell-based, BMP-activity assays, when B-17 is added in excess to BMP-2 there is no inhibition of activity (data not shown). BC-1 increases the retention of BMP-2 in the collagen matrix and leads to increases in osteogenic activity and bone formation but whether the BMP-2 is free or peptide-bound is not clear at this point.

One of the challenges of local delivery of growth factors is the retention of the growth factor at the site of healing for a sufficient time to allow the growth factor to have a positive effect. An FDA–approved BMP-delivery system uses a collagen matrix and requires supra-physiologic doses of BMP to achieve the desired therapeutic effect which increases concerns about safety and cost [24]. Collagen matrices have many desirable features in growth factor delivery systems including biocompatibility, degradation to natural products and favorable interactions with cells; but when used with BMPs, collagen matrices rapidly lose BMP after implantation [23]. Using an absorbable collagen sponge (ACS), 30% of the loaded BMP-2 is lost almost immediately and 50% is lost in the first two days after implantation [23]. Incorporation of a bifunctional collagen: growth factor–binding peptide as described here could greatly enhance the performance of collagen-based drug delivery systems. In addition, the ability of these BMP-2 binding peptides to bind other members of the BMP family, such as BMP-7, extends their utility to other BMPs.

An alternative to a bifunctional peptide is to covalently attach the BMP-binding peptide directly to the collagen matrix. In fact, some of us have done this (HG, SG, MJ, SN, JG and JH; manuscript in preparation). Covalent attachment of peptides to a matrix such as collagen requires a series of chemical treatments and can alter the handling and performance characteristics of the matrix. However, eliminating the matrix-binding domain and covalently attaching the BMP-binding peptide directly to the matrix can increase the apparent affinity of the matrix for the BMP compared to using an equimolar amount of bifunctional peptide mixed with matrix. The advantage of a bifunctional peptide is that it requires no modification of the matrix and allows for a “mix and use” situation.

Growth factors have enormous potential as biopharmaceuticals used in tissue and organ repair. Effective utilization, however, will require delivery systems that can target the release to a specific site and control the dose to enhance the healing response with minimal activity away from the site of repair. The collagen-BMP bifunctional peptide described in this paper has the potential to enhance healing of bone with a targeted, controlled delivery of BMP-2 from an injectable collagen matrix. Similarly, growth factor–binding peptides could be incorporated into a variety of site-specific delivery systems where a localized, controlled-release of growth factor is required.
